# The Predictive Value of the CSA Index in the Prenatal Diagnosis of Aortic Coarctation in Ultrasound Examination Performed during the Second Trimester

**DOI:** 10.3390/jcm12165190

**Published:** 2023-08-09

**Authors:** Katarzyna Zych-Krekora, Michał Krekora, Mariusz Grzesiak, Oskar Sylwestrzak

**Affiliations:** 1 Department of Perinatology, Obstetrics and Gynecology, Polish Mother’s Memorial Hospital Research Institute in Lodz, 93-338 Łódź, Poland; mariusz.grzesiak@gmail.com; 2 Department of Obstetrics and Gynecology, Polish Mother’s Memorial Hospital Research Institute in Lodz, 93-338 Łódź, Poland; krekoram@poczta.onet.pl (M.K.); sylwestrzakoskarpatryk@gmail.com (O.S.); 3 Department of Gynecology and Obstetrics, Medical University of Lodz, 90-419 Łódź, Poland; 4 Department of Prenatal Cardiology, Polish Mother’s Memorial Hospital Research Institute in Lodz, 93-338 Łódź, Poland

**Keywords:** fetal echocardiography, congenital heart defect, coarctation of the aorta

## Abstract

Background: Aortic coarctation (CoA) is the fourth most common congenital heart defect (8–10%) which occurs at a frequency of about 20–60/100,000 births. Only 22.3% of all cases appears to be diagnosed during the second trimester of pregnancy. Since the detection of prenatal aortic coarctations is very low, every effort should be made to change this situation. According to the authors of this study, the CSAi (carotid to subclavian artery index) could serve as a reliable indicator. Material and Methods: Ninety-six fetuses from healthy, single, pregnancies, with good ultrasound visualization between 18 and 27.5 weeks of gestation, and twenty-three fetuses suspected of aortic coarctation (postnatally confirmed) were included in this study. Our first aim was to compare the current most common method of prenatal CoA diagnosis based on the measurement of the aortic z-score in the aortic isthmus using the method suggested by us-CSAi. Results: Logistic regression coefficients for z-score and CSAi were analyzed as predictors of coarctation occurrence. It appears that 39.4% of coarctation occurrence can be predicted on the basis of the z-score, and 93.5% on the basis of the CSAi. The cut-off value for CSAi in the study group was 0.81 (sensitivity: 95.7%, specificity 99%). Based on the ROC curve analysis, the cut-off value for the carotid to subclavian distance (mm) was determined; the risk of coarctation increased above this value. Based on the Gini index (0.867), this value was set at 2.55 (sensitivity 82.6%, specificity 93.7%). Conclusions: CSAi measurement is currently the most sensitive method for aortic coarctation detection. For the purpose of our study, this method was applied in diagnostics in the second trimester of pregnancy. This method is easy, reproducible and should be widely introduced into everyday echocardiographic diagnostics of coarctation to minimize the risk of error.

## 1. Introduction

Aortic coarctation (CoA) is considered the fourth most common congenital heart defect, accounting for approximately 8–10% of cases. Its occurrence rate is estimated to be between 20 and 60 per 100,000 births, with a higher prevalence in males compared to females. Additionally, children with chromosomal aberrations are more susceptible to developing CoA [1]. The severity of symptoms experienced by individuals with this condition is contingent upon the degree of coarctation. The defect’s dependence on the flow in the Botallo’s arterial duct underscores the significance of prompt postnatal diagnosis, as delayed detection can adversely affect the prognosis. In newborns, the manifestation of the defect can be severe and life-threatening. Failure to treat CoA can lead to the development of heart failure, metabolic acidosis, anuria, and shock. While the primary manifestations of the disease are observed during the neonatal period, most patients tend to be asymptomatic in subsequent stages. Surprisingly, up to 25% of aortic coarctation cases are diagnosed after the age of 10, and sometimes even in adulthood [2]. Over a span of several years, the heart defect induces irreversible changes, in particular, persistent arterial hypertension. It is essential to note that aortic coarctation is not a homogeneous group of defects, as approximately 85% of children with CoA also exhibit a bicuspid aortic valve. The diagnosis of fetal coarctation continues to pose challenges for physicians performing prenatal ultrasound. Historically, the presence of coarctation has been suspected primarily based on ventricular disproportion, with the left ventricle appearing smaller than the right. However, this approach demonstrates only moderate sensitivity and a high rate of false-positive results [3]. Consequently, there is a pressing need to explore more sensitive and specific methods for diagnosing this heart defect. Furthermore, it is crucial to underscore the importance of prenatal diagnosis for coarctation, considering its potential for a severe postnatal course. Therefore, childbirth should be planned in a third-level reference center, ensuring access to multidisciplinary care and cardiological/cardiac surgery therapy for the child. The main aim of this study was to compare aortic arch z-score measurements with the carotid to subclavian artery index (CSAi) for their effectiveness in predicting CoA prenatally.

## 2. Materials and Methods

Echocardiographic examinations were conducted at the Department of Obstetrics, Perinatology, and Gynecology of the Institute of Polish Mother’s Health Centre in Łódź, by physicians (KZK) who were qualified in fetal echocardiography. In this study, we included a total of 96 fetuses from healthy, single pregnancies with good ultrasound visualization between 18 and 27.5 weeks of gestation. Additionally, 23 fetuses suspected of aortic coarctation, which were later confirmed postnatally, were also included.

Our primary objective was to compare the current, most common method of prenatal CoA diagnosis, which is based on the measurement of the aortic z-score in the aortic isthmus, with the method proposed by our team. Our method utilizes the CSAi, which is calculated based on the distance between the carotid and subclavian artery in longitudinal visualization, as well as the transverse dimension of the aorta between these two vessels. The calculation formula for CSAi is as follows:

CSAi = Ao transverse diameter (mm)/carotid to subclavian diameter (mm)

**Figure 1:**
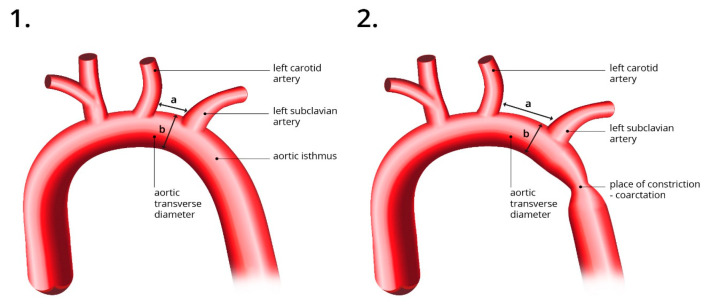
The figure on the left (**1**) illustrates a normal aortic arch with CSAi measurement and a normal aortic isthmus visible. The right figure (**2**) shows an elongated distance between the carotid and subclavian arteries and the site of stenosis-coarctation. a—the distance between the carotid and subclavian artery; b—Ao transverse diameter.

**Figure 2:**
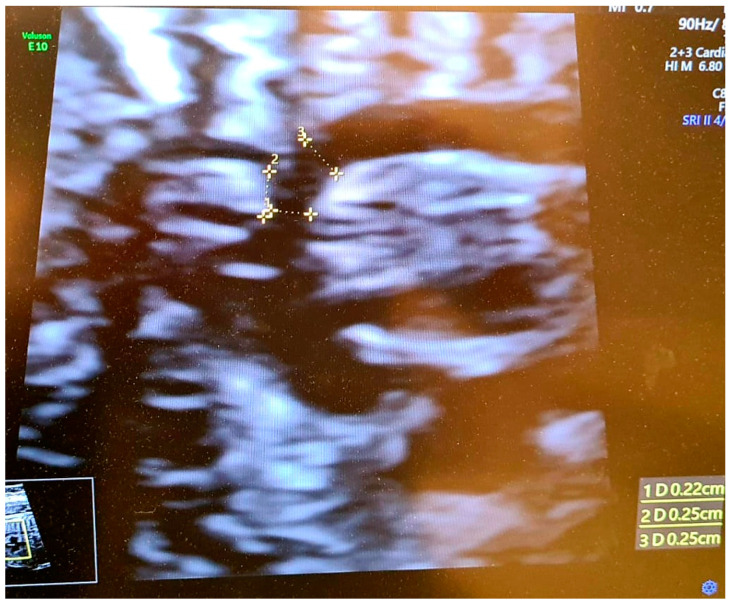
Ultrasound view: Correct visualization of the aortic arch at 23.5 week of pregnancy—with visible brachiocephalic trunk. The dimension from carotid distance to subclavian (2 D) = 2.5 mm, transverse aortic dimension (1 D) = 2.2 mm, calculated CSAi = 0.88. Aortic isthmus (3 D) 2.5 mm, z-score = −0.58.

To ensure accuracy in the measurement process, we have provided a visual representation of the correct method of measurement, which can be found in Figure 1 and Figure 2.

The comparison between the two diagnostic methods, namely the aortic z-score and CSAi, aims to evaluate their effectiveness in prenatal CoA diagnosis. By conducting this comparative analysis, we aim to determine if the CSAi method offers any advantages over the current standard approach. This research endeavor will contribute valuable insights to improve the accuracy and reliability of prenatal diagnosis for aortic coarctation, ultimately enhancing patient care and outcomes.

Statistical analyses were performed with the use of the Jamovi 2.3.2 program. The normal distribution of variables was verified using the Shapiro–Wilk test. To compare the control and coarctation groups in terms of the values of the CSAi and z-score (Pasquini L 2007) [4], the Mann–Whitney U test was used (due to significant disproportions in the size of the compared groups). Incorrect z-score results were defined as equal to or less than −2.0, or equal to or greater than 2.0. Logistic regression analysis was performed to determine which of the indexes better predicted the occurrence of coarctation. Moreover, the sensitivity and specificity as well as the accuracy of the predictions were established, based on the ROC curve. Pearson’s correlation coefficient (r) was used to find associations between gestational age and z-scores and CSAI. The level of significance was α = 0.05.

## 3. Results

Table 1 presents the basic descriptive statistics for gestational age, CSAi, and z-scores in both the control and coarctation groups. The Shapiro–Wilk test was conducted to assess the normality of the variable distributions.

In the control group (N = 96), the mean gestational age was 21.29 weeks (SD = 1.71), indicating a relatively consistent age range. The gestational age variable had a normal distribution (W = 0.94, *p* < 0.001), implying that the data points were well-distributed around the mean. The z-score variable had a mean of −0.50 (SD = 1.06), suggesting a slight deviation from the expected value. The CSAi variable had a mean of 1.24 (SD = 0.32), indicating the average value of the index in the control group.

On the other hand, in the coarctation group (N = 23), the mean gestational age was 23.06 weeks (SD = 2.45). The Shapiro–Wilk test result for gestational age showed that the variable also followed a normal distribution (W = 0.98, *p* = 0.874). The z-score variable had a mean of −3.06 (SD = 1.92), indicating a significant deviation from the expected value. However, the Shapiro–Wilk test result for the z-score did not reach statistical significance (W = 0.92, *p* = 0.056). The CSAi variable had a mean of 0.49 (SD = 0.16), suggesting the average value of the index in the coarctation group. The Shapiro–Wilk test result for CSAi indicated a normal distribution (W = 0.97, *p* = 0.733).

These results demonstrate that the variables in the control group, including gestational age, z-scores, and CSAi, followed a normal distribution. However, in the coarctation group, while the gestational age and CSAi variables exhibited normal distributions, the z-score variable showed a slightly skewed distribution, albeit not reaching statistical significance.

**Figure 3:**
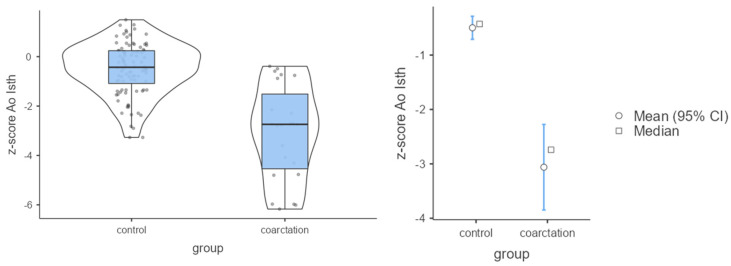
Mean values of Ao Isth and Z-scores in the control and coarctation groups.

**Figure 4:**
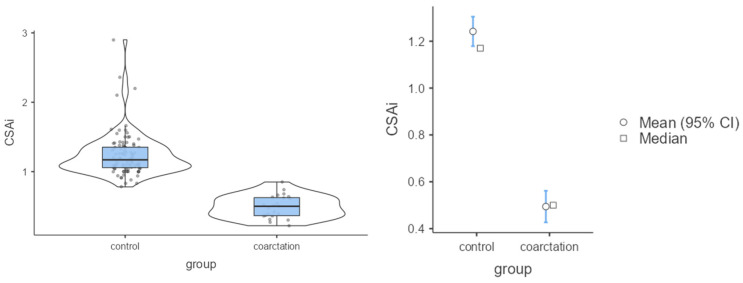
Mean values of CSAi in the control group and group with coarctation.

The analysis showed that in the control group both the CSAi index and the z-score had higher values compared with the coarctation group (*p* < 0.001). Because of these differences, the strength of the effect was high (Table 2; Figure 3 and Figure 4). In the control group, the percentage of incorrect Z-scores was 10.4% (n = 10), while in the coarctation group it was higher and amounted to 73.9% (n = 17).

Table 3 provides the logistic regression coefficients for z-score and CSAi as predictors of coarctation occurrence. The results indicate that the z-score can predict approximately 39.4% of coarctation cases, while the CSAi index demonstrates a higher predictive capability of 93.5%.

The logistic regression coefficients reveal that as the z-score increases, there is a significant decrease in the probability of coarctation occurrence (odds ratio (OR) = 0.30; 95% confidence interval (CI) 0.18–0.49). This means that for every unit increase in the z-score, the likelihood of coarctation decreases by a factor of 0.30, within the specified confidence interval.

Similarly, with an increase in the CSAi index, the probability of coarctation occurrence decreases even further (OR < 0.01; 95% CI < 0.01–0.02). This suggests that for every unit increase in the CSAi index, the odds of coarctation occurring decrease to a negligible value, according to the odds ratio within the given confidence interval.

These findings demonstrate that both the z-score and CSAi index hold significant predictive value for coarctation occurrence. The higher predictability observed with the CSAi index emphasizes its superiority in identifying cases of coarctation. Conversely, the z-score, while still providing valuable predictive information, is less robust compared to the CSAi index. These results provide important insights into the association between these predictors and the probability of coarctation occurrence, offering clinicians valuable tools for risk assessment and decision-making in the diagnosis and management of this condition.

**Figure 5:**
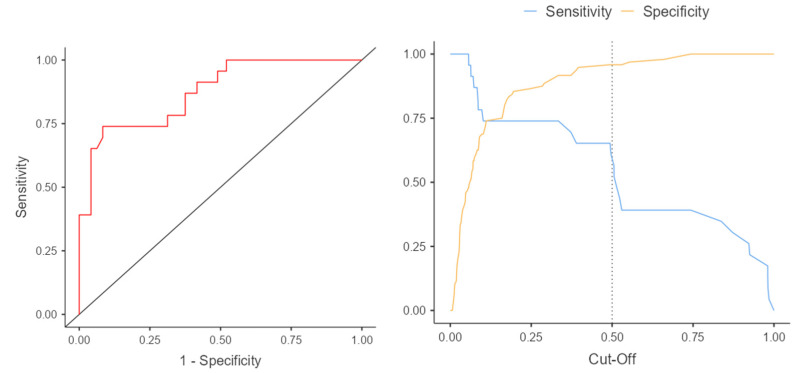
ROC curve and cut-off points for the z-score.

**Figure 6:**
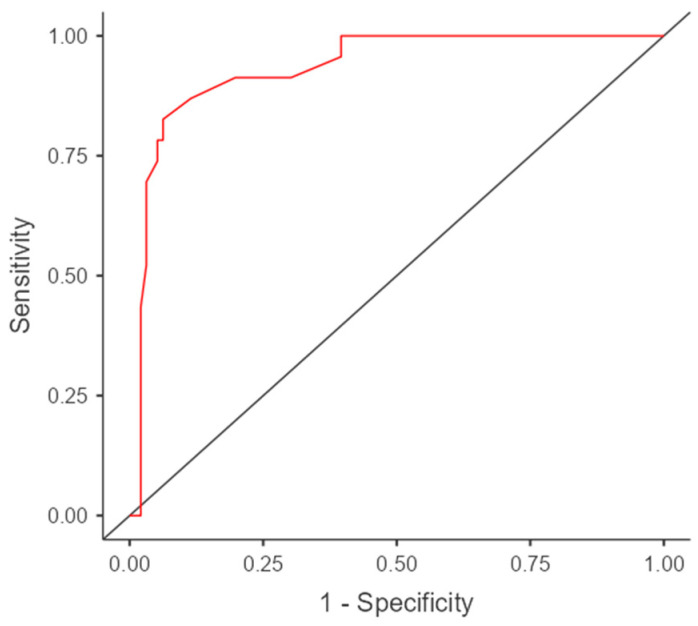
The cut-off value for carotid to subclavian distance (mm), above which the risk of coarctation increases.

In the case of classifying into the coarctation group based on the z-score, the accuracy was 56.5%, whereas when using the CSAi index, it was 95.7%. Predicting the occurrence of aortic coarctation based on the CSAi index proved to be more precise than using the z-score (Table 4). The ROC curves and intersection point graphs for both indices are presented in Figure 5 and Figure 6.

The results of the ROC analysis and ROC curves confirm the differences in predictive accuracy between the z-score and CSAi in diagnosing aortic coarctation. The ROC curve for the z-score presents sensitivity and specificity values for various thresholds, while the ROC curve for CSAi illustrates the sensitivity and specificity values for different index values. The intersection point of the curves, known as the cutoff point, corresponds to the value above which the risk of aortic coarctation occurrence is considered significant.

Analyzing these curves and the intersection point enables the selection of an appropriate diagnostic threshold that minimizes classification errors and maximizes diagnostic accuracy in the prenatal detection of aortic coarctation.

The results of the analysis using Pearson’s correlation showed weak and negative correlations between gestational age and z-scores (r = −0.20; *p* = 0.032) as well as between gestational age and CSAi (r = −0.25; *p* = 0.006). This means that as the pregnancy progresses, both the z-scores and CSAi tend to decrease, indicating a lower value of these indexes in more advanced pregnancies.

To further evaluate the diagnostic performance of the carotid to subclavian distance in predicting coarctation, a receiver operating characteristic (ROC) curve analysis was conducted. The cut-off value for the distance was determined based on the ROC curve, which represents the trade-off between sensitivity and specificity. The Gini index, which measures the discriminatory power of the model, was calculated to be 0.867, indicating a strong discriminatory ability.

The cut-off value for the carotid to subclavian distance was determined to be 2.55 mm. When the distance exceeded this value, the risk of coarctation was found to increase. The sensitivity of the cut-off value was calculated to be 82.6%, meaning that it correctly identified 82.6% of cases with coarctation. The specificity, which indicates the ability to correctly identify cases without coarctation, was determined to be 93.7%.

The area under the ROC curve (AUC), which provides an overall measure of the diagnostic accuracy of the test, was calculated to be 0.933 with a 95% confidence interval of 0.88–0.99. An AUC value close to 1 indicates a high accuracy of the test in distinguishing between individuals with and without the condition.

These findings suggest that the carotid to subclavian distance can serve as a reliable indicator for predicting coarctation, with a significant association between the distance and the presence of the condition. The cut-off value determined provides a threshold above which the risk of coarctation increases, aiding in the prenatal diagnosis and management of this heart defect.

## 4. Discussion

CoA is generally well tolerated during fetal life. The fetal cardiovascular system profoundly differs from that of the neonatal, as the right ventricle is the systemic chamber and generates more cardiac output. The situation changes after birth. Rapid deterioration may occur after ductus arteriosus closure and the degree of heart failure varies depending on the severity of the aortic narrowing [5]. Cases with a less narrow CoA can be asymptomatic after birth and remain asymptomatic until hypertension occurs. Arterial collateral vessels could develop and bypass aortic narrowing [6]. CoA requires interventional treatment. The management differs and depends on the severity and clinical status of the patient. Balloon angioplasty, aortic arch stenting or cardiosurgery may be performed, but even if treatment was introduced in time, patients with a neonatal CoA repair are at risk of adverse neurodevelopmental outcomes and these patients could benefit from timely neurocognitive evaluation and intervention [7].

Fetal CoA is associated with subtle and inconsistent features in the mid-trimester; that is why CoA is sometimes called the Achilles heel of fetal cardiology. It is also the most common ductal-dependent lesion missed on neonatal exam screening [8]. There are many false-positive and false-negative results in general practice. What is more, CoA is often a progressive lesion and, at late gestation, its features may resemble normal physiological changes in the fetal hemodynamic [9]. Both false-negative and false-positive prenatal diagnosis of CoA could have a disadvantageous impact on postnatal management. False-positive diagnosis alter birth location and could lead to unnecessary management and finally generate unnecessary costs. On the other hand, false-negative results may cause neonatal cardiovascular deterioration, hypoxia and following complications. To enhance the diagnosis of fetal CoA, 3-dimensional magnetic resonance (MRI) was investigated by Lloyd et al. [10]. Nevertheless, MRI is much more expensive than fetal echocardiography and not so widely used. Nowadays significant advancements in prenatal ultrasound diagnostics have been made and still clinicians’ abilities are increasing. However, only about 22% of cases are typically identified during the second trimester of pregnancy and more than 50% of aortic coarctation cases are diagnosed by neonatologists [11,12]. This imposes a strong need to develop a fetal echocardiographic method to predict CoA after birth. A prenatal diagnosis of CoA reduces the mortality and morbidity associated with this defect and the postnatal outcome is significantly better when prenatal detection is not missed [13]. 

Currently, the most common method used to detect coarctation is by assessing ventricular dimensions and identifying disproportions between the right and left ventricles (R > L). However, this approach has moderate sensitivity in diagnosing coarctation and an alarmingly high rate of false positives, reaching up to 80% [14,15]. Another diagnostic method involves assessing the disproportions between the great arterial trunks at the level of the superior mediastinum, just behind the sternum and thymus. Researchers have analyzed the usefulness of measuring the diameters of the main pulmonary artery (MPA) and aorta (Ao) in the third trimester, particularly the MPA/Ao diameter ratio. A ratio of 1.60 or greater has shown a sensitivity of 83%, specificity of 85%, and a positive predictive value of 62.5% in distinguishing between true and false coarctations [16]. Although this method is an improvement, its usefulness is limited to the third trimester, where the conditions for echocardiography examination are already significantly restricted due to advanced gestational age, bone tissue calcification, acoustic shadow, and the fetus frequently being positioned with its back to the examiner.

In 2007, Pasquini et al. developed scores for the aortic isthmus in healthy fetuses as a reference point for suspected coarctation cases. These scores established z-scores, which relate the diameter of the isthmus and ductus to the length of the femur and gestational age. While z-scores have proven helpful in diagnosing aortic defects, they cannot be solely relied upon for diagnosis due to clinically significant and unacceptable differences in z-scores across different ultrasound machines and examiners [4].

To improve the prenatal detection rate of aortic coarctation, efforts should be made to find more reliable indicators. CSAi has shown promise in the postnatal detection of coarctation in newborns and infants, demonstrating a positive predictive value of 97.7% and 100%, respectively [17]. Subsequent studies in newborns have confirmed the high predictability of CoA diagnosis using CSAi, with a CSAi value < 1 correlating with the presence of coarctation in almost 92% of patients [18].

In 2021, a published article further confirmed the usefulness of CSAi in predicting CoA in fetuses. The study included 65 fetuses with prenatal suspicion of isolated CoA and found that a CSAi < 0.78 had a sensitivity of 92.3% and specificity of 96.8% in predicting CoA [19]. However, it is essential to consider that this study focused on fetuses with a mean gestational age of 34.1 weeks at the time of echocardiography.

In the study by Wang et al., logistic regression analysis was conducted to develop a probability model for the prenatal diagnosis of CoA [20]. The model incorporated prenatal cardiac sonographic markers and achieved an optimal criterion of >0.25, resulting in a sensitivity of 97.7% and specificity of 59.1%. The parameters used in the model were the left common carotid artery to left subclavian artery distance divided by the distal transverse arch (LCCA-LSCA/DT index) and the z-score of the peak Doppler flow in the aorta. The authors found that the probability model performed well in predicting CoA outcomes after birth and improved the accuracy of risk assessment. They proposed that fetuses with a model probability > 60% should undergo inpatient observation due to a high risk of CoA, while those with a model probability < 15% should not require clinical follow-up. 

On the other hand, Bartolacelli et al.aimed to develop an echocardiographic pre-diction model for newborns with a prenatal suspicion of CoA who still had a patent ductus arteriosus (PDA) at birth, to predict the need for surgical intervention [21]. In a retrospective study, they included term and late preterm newborns with prenatal suspicion of CoA born between 2007 and 2020. The predictive model (CoMOD) included the dimensions of the aortic isthmus, transverse arch, the distance between the left common carotid artery and left subclavian artery, and the presence or absence of ventricular septal defect and bicuspid aortic valve. The results showed that CoMOD had an AUC of 0.9382, high sensitivity (91%), and specificity (86%) in predicting the need for surgical correction of CoA in newborns with prenatal suspicion. The authors suggested that newborns with a CoMOD value >0 were highly likely to require surgical repair of CoA, with a good positive predictive value (PPV) of 86.9% and negative predictive value (NPV) of 90.9%.

Comparing both studies, it can be observed that both CSAi and CoMOD are promising tools in the prediction of fetal aortic coarctation. CSAi was analyzed in the study by Wang et al., demonstrating high effectiveness in predicting CoA. On the other hand, CoMOD was developed in the study by Bartolacelli et al., also showing high sensitivity and specificity in predicting the need for surgical intervention in newborns with a prenatal suspicion of CoA.

These findings suggest that both CSAi and CoMOD can be useful in prenatal diagnosis of aortic coarctation, improving the accuracy of risk assessment and enabling better patient management. Implementing these methods in routine echocardiographic diagnostics may contribute to reducing diagnostic errors and improving treatment outcomes for patients with CoA.

The standardization of parameters across ultrasound machines is crucial for obtaining reliable z-scores in fetal echocardiography. Currently, there are significant discrepancies in z-scores developed by different ultrasound examiners and available machines. Cooperation between multiple centers is necessary to establish standardized parameters for all ultrasound machines and improve the accuracy of z-scores.

In summary, CSAi has emerged as the more reliable predictor of aortic coarctation, starting from the second trimester of pregnancy, with a high sensitivity and specificity. The cut-off values for CSAi and the assessment of the distance between the carotid and subclavian arteries should be incorporated into routine echocardiographic diagnostics to minimize diagnostic errors. However, caution should be exercised when relying solely on aortic z-scores due to discrepancies across different ultrasound machines and examiners.

## 5. Conclusions

Based on our research, we observed that CSAi is currently the most reliable predictor of aortic coarctation starting from the second trimester of pregnancy (93%). The cut-off value for CSAi in the study group was 0.81 (sensitivity: 95.7%, specificity 99%). At the beginning of the examination, the measurement of the CSAi index can be supplemented by the assessment of the distance between the carotid and subclavian arteries. In this case, the cut-off point in the study group was set at 2.55 mm (with a lower sensitivity of 82.6% and a slightly lower specificity of 93.7%). Due to large discrepancies in the aortic z-score evaluated by different authors, this indicator cannot be commonly used in the diagnosis of coarctation. Our research confirmed that its predictive value is only 39.4% (sensitivity 56%, specificity 95%). CSAi is easy, reproducible and should be widely introduced into everyday echocardiographic diagnostics of coarctation to minimize the risk of error.

## Figures and Tables

**Table 1:** Descriptive statistics.

	M	Me	SD	Sk.	Kurt.	Min.	Max.	W	*p*
**Control (N = 96)**									
Weeks	21.29	21.10	1.71	0.63	0.01	18.00	25.40	0.94	<0.001
Z-score	−0.50	−0.43	1.06	−0.55	−0.06	−3.27	1.49	0.97	0.033
CSAI	1.24	1.17	0.32	2.46	9.23	0.78	2.90	0.80	<0.001
**Coarctation (N = 23)**									
Weeks	23.06	23.00	2.45	0.02	−0.33	18.20	27.50	0.98	0.874
Z-score	−3.06	−2.74	1.92	−0.21	−1.08	−6.17	−0.39	0.92	0.056
CSAI	0.49	0.50	0.16	0.28	−0.64	0.22	0.85	0.97	0.733

M—mean; Me—median; SD—standard deviation; Sk.—skewness; Kurt.—kurtosis; W—Shapiro–Wilk test result; *p*—significance.

**Table 2:** The comparison of the control group and the coarctation group in terms of z-score and CSAi values.

	Control (n = 96)			Coarctation (n = 23)					
	Mean Rank	Me	IQR	Mean Rank	Me	IQR	Z	*p*	R
z-score Ao Isth	68.60	−0.43	1.50	24.09	−2.74	3.89	−5.56	<0.001	0.51
CSAi	71.47	1.17	0.31	12.13	0.50	0.27	−7.41	<0.001	0.68

**Table 3:** Logistic regression coefficients for the prediction of the occurrence of coarctation.

	B	SE	Z	*p*	OR	95% CI	R2MCF
z-score	−1.21	0.25	−4.77	<0.001	0.30	0.18; 0.49	0.394
CSAI	−30.40	13.60	−2.24	0.025	<0.01	<0.01; 0.02	0.935

**Table 4:** Summary of the quality of coarctation prediction based on z-scores and CSAi.

Index	Accuracy	Specificity	Sensitivity	AUC
z-score	0.882	0.958	0.565	0.874
CSAI	0.983	0.990	0.957	0.999

## Data Availability

Not applicable.
